# Venous thromboembolism chemical prophylaxis after endoscopic trans-sphenoidal pituitary surgery

**DOI:** 10.1007/s11102-021-01195-8

**Published:** 2021-11-29

**Authors:** Mueez Waqar, Annabel Chadwick, James Kersey, Daniel Horner, Tara Kearney, Konstantina Karabatsou, Kanna K. Gnanalingham, Omar N. Pathmanaban

**Affiliations:** 1 Department of Neurosurgery, Manchester Centre for Clinical Neurosciences, Salford Royal NHS Foundation Trust, Manchester, UK; 2 Faculty of Biology, Medicine and Health, Geoffrey Jefferson Brain Research Centre, The University of Manchester, Manchester, UK; 3 Department of Neurocritical Care, Manchester Centre for Clinical Neurosciences, Salford Royal NHS Foundation Trust, Manchester, UK; 4 Department of Endocrinology, Manchester Centre for Clinical Neurosciences, Salford Royal NHS Foundation Trust, Manchester, UK; 5 School of Medicine, Geoffrey Jefferson Brain Research Centre, The University of Manchester, Manchester, UK

**Keywords:** Venous thromboembolism, VTE, Hematoma, Haematoma, Transsphenoidal, Pituitary, Heparin, Prophylaxis, Chemoprophylaxis

## Abstract

**Purpose:**

There is no compelling outcome data or clear guidance surrounding postoperative venous thromboembolism (VTE) prophylaxis using low molecular weight heparin (chemoprophylaxis) in patients undergoing pituitary surgery. Here we describe our experience of early chemoprophylaxis (post-operative day 1) following trans-sphenoidal pituitary surgery.

**Methods:**

Single-centre review of a prospective surgical database and VTE records. Adults undergoing first time trans-sphenoidal pituitary surgery were included (2009–2018). VTE was defined as either deep vein thrombosis and/or pulmonary embolism within 3 months of surgery. Postoperative haematomas were those associated with a clinical deterioration together with radiological evidence.

**Results:**

651 Patients included with a median age of 55 years (range 16–86 years). Most (99%) patients underwent trans-sphenoidal surgery using a standard endoscopic single nostril or bi-nostril trans-sphenoidal technique. More than three quarters had pituitary adenomas (n = 520, 80%). Postoperative chemoprophylaxis to prevent VTE was administered in 478 patients (73%). Chemoprophylaxis was initiated at a median of 1 day post-procedure (range 1–5 days postoperatively; 92% on postoperative day 1). Tinzaparin was used in 465/478 patients (97%) and enoxaparin was used in 14/478 (3%). There were no cases of VTE, even in 78 ACTH-dependent Cushing’s disease patients. Six patients (1%) developed postoperative haematomas. Chemoprophylaxis was not associated with a significantly higher rate of postoperative haematoma formation (Fisher’s Exact, p = 0.99) or epistaxis (Fisher’s Exact, p > 0.99).

**Conclusions:**

Chemoprophylaxis after trans-sphenoidal pituitary surgery on post-operative day 1 is a safe strategy to reduce the risk of VTE without significantly increasing the risk of postoperative bleeding events.

## Introduction

Venous thromboembolism (VTE) is the composite of deep vein thrombosis and pulmonary embolism, which are serious and potentially fatal complications of neurosurgery. Such complications are potentially preventable. The risk of VTE can be reduced through mechanical thromboprophylaxis with compression stockings and intermittent pneumatic compression, along with pharmacological thromboprophylaxis (chemoprophylaxis). These measures can be used in conjunction. However, there is limited evidence of benefit for mechanical thromboprophylaxis in patients receiving pharmacological agents and for those patients who are critically ill [1, 2]. There is strong evidence of benefit for pharmacological prophylaxis in hospitalised patients [3], though data for patients undergoing cranial neurosurgery is limited.

The American Heart Association recommends against using chemoprophylaxis in patients undergoing major neurosurgical procedures, except in those deemed at high risk, such as those experiencing prolonged immobility [4]. These guidelines are open to interpretation and do not specifically address pituitary surgery, which is also the case for the European guidelines [5]. The National Institute for Health and Care Excellence (NICE) suggest initiation of chemoprophylaxis 24–48 h after cranial neurosurgery where benefits outweigh risks [6]. As such, decision making surrounding chemoprophylaxis must be made on a case by case basis by balancing the benefits of VTE reduction with potentially increased risk of bleeding events. Meta-analyses and prior institutional series including neurosurgical patients from different subspecialties have demonstrated that pharmacological thromboprophylaxis with heparin agents can be used postoperatively to decrease the rate of VTE with a minimal risk of bleeding [7–12]. However, there is a lack of subset outcome data for patients undergoing trans-sphenoidal pituitary surgery. In this cohort of patients, the potential risks of chemoprophylaxis include postoperative haematoma with the potential for serious sequelae including acute visual failure, hypopituitarism, hypothalamic compromise and hydrocephalus.

Although rates of VTE following pituitary surgery have previously been identified as being relatively low, several risk factors have been identified including increasing age, peripheral vascular disease and coagulopathy [13]. Furthermore, patients with Cushing’s disease may be at an increased risk of VTE both prior to and following tumour resection [14, 15]. Data in the specific context of trans-sphenoidal pituitary surgery is therefore important. The aim of this study was to describe our experience of chemoprophylaxis following trans-sphenoidal pituitary surgery and identify the rate of relevant outcomes, such as VTE and clinically significant bleeding events including epistaxis and post-operative haematoma formation.

## Methods

Institutional Review Board approval was obtained for this single-centre observational study. We interrogated a prospectively maintained electronic database of locally performed neurosurgical operations. All adults (age ≥ 16 years) undergoing first time endoscopic trans-sphenoidal pituitary surgery between April 2009 and November 2018 were included. Patients with redo operations were excluded. All procedures were performed by the three senior authors (OP, KG, KK).

The electronic patient record was reviewed and data extracted on demographics, histological diagnosis, operative intervention, postoperative course (including use of chemoprophylaxis) and the development of VTE or clinically significant bleeding events including epistaxis and postoperative haematomas. In accordance with national standards and the NHS contract, the trust thrombosis committee maintains a separate electronic and prospectively maintained database of VTE positive events occurring during hospital admission or within 90 days of hospital discharge, following inpatient admission (hospital acquired thrombosis). This dataset was cross referenced to identify any missed VTE events within the included cohort.

Our surgical technique has been previous described [16]. Antiplatelet medication is discontinued 5 prior to surgery. Anticoagulant medication is discontinued 5 days (warfarin) or 48 h (novel oral anticoagulants) before surgery. In general, a standard trans-nasal, trans-sphenoidal approach is utilised with a single nostril or bi-nostril modified Griffiths technique. Postoperatively, patients are monitored in a high dependency unit for at least 24 h. Patients are encouraged to mobilise postoperatively and there are no bed rest restrictions. Within the study period, there was a change in practice to early postoperative chemoprophylaxis, usually from the first postoperative day. The decision to commence postoperative chemoprophylaxis is made after the procedure on daily patient rounds. Potential factors that can influence this decision and the exact day of commencement (e.g. postoperative day 1 vs. subsequent days vs. not at all) include the degree of difficulty encountered with intraoperative haemostasis, coagulation state, preoperative comorbidities and antiplatelets/anticoagulant use, and patient mobility.

The preferred low molecular weight heparin (LMWH) agent at our institute is tinzaparin, with dose adjustment to weight and renal function. Chemoprophylaxis is used together with mechanical prophylaxis at our institute; all patients had mechanical prophylaxis with Flowtron® boots (intraoperatively) and TED stockings from hospital admission to discharge as per our institutional protocol. Chemoprophylaxis was discontinued on the day of discharge, with no routine discharge course.

Imaging is not routinely performed in the immediate postoperative period unless clinically indicated. Therefore, postoperative haematomas described in the present study were clinically detected with a deterioration in vision or neurology postoperatively and demonstrated on subsequent magnetic resonance imaging (MRI) or computed tomography (CT) scans.

Statistical analysis was performed using SPSS version 25 (SPSS, Inc., Chicago, Illinois, USA). Descriptive statistics were used to describe patient cohort characteristics. Categorical variables were compared using tests of proportions (Fisher’s Exact, Chi-squared). Multivariate analysis was not possible due to the low incidence of haematomas.

## Results

### Patient characteristics

651 Patients were included. The median age at the time of surgery was 55 years (range 16–86 years). There were equal numbers of males (n = 326, 50%) and females (n = 325, 50%). 39 Patients (6%) were taking antiplatelet medication prior to surgery and 14 (2%) were taking anticoagulants that were discontinued before surgery. Further baseline demographic details are provided in Table 1.

**Table 1:** Patient characteristics

Age (years)	
Median	55
Range	15–86
Gender	
Male	326 (50%)
Female	325 (50%)
Antiplatelets	
Total	39 (6%)
Aspirin	29 (4%)
Clopidogrel	10 (2%)
Anticoagulants	
Total	14 (2%)
Warfarin	8 (1%)
Heparin	3 (< 1%)
NOAC	3 (< 1%)
Diabetes mellitus	159 (24%)
Thrombocytopenia	18 (3%)
Histology	
Pituitary adenoma	520 (80%)
NFPA/gonadotroph adenoma	308 (47%)
Corticotroph adenoma	79 (12%)
GH adenoma	104 (16%)
TSH adenoma	6 (1%)
Prolactinoma	8 (1%)
Plurihormonal/other adenoma	15 (2%)
Rathke's cyst	20 (3%)
Craniopharyngioma	13 (2%)
Other tumour*	21 (3%)
Cystic lesion	12 (2%)
Inflammatory/infective	24 (4%)
Vascular (apoplexy)	17 (3%)
Non-diagnostic	24 (4%)

*Includes metastases, lymphoma, myeloma, and neurocytoma

Most patients (646, 99%) underwent trans-sphenoidal surgery using a standard technique. An extended trans-sphenoidal approach was used in 5 patients (1%).

Histology breakdowns are provided in Table 1. More than three quarters of patients had pituitary adenomas (n = 520, 80%). The commonest subtypes were the non-functioning pituitary adenomas (NFPAs)/gonadotroph adenomas (n = 308, 47%). 79 Patients (12%) had ACTH-dependent Cushing’s disease and 104 patients (16%) had acromegaly.

### Chemoprophylaxis

Postoperative chemoprophylaxis to prevent VTE was administered in 478/651 patients (73%). Tinzaparin was used in 465/478 patients (97%) and enoxaparin was used in 14/478 (3%). The median dose of tinzaparin was 4500 units (range 2500–18,000 units). The median dose of enoxaparin was 40 mg (range 20–40 mg).

Chemoprophylaxis was initiated at a median of 1 day post-procedure (range 1–5 days postoperatively). Early chemoprophylaxis on the first postoperative day was used in 439/478 patients (92%). The early chemoprophylaxis group included 4 out of 5 patients that underwent an extended trans-sphenoidal approach, with the remaining patient not receiving chemoprophylaxis. Delayed chemoprophylaxis was used in 39/478 patients (8%). The median duration of chemoprophylaxis was 2 days (range 1–53 days). Only 7/465 patients (2%) had chemoprophylaxis used for longer than 2 weeks. Chemoprophylaxis was used exclusively in the hospital setting and no patients had continuation upon discharge.

A comparison of characteristics between patients that did/did not receive chemoprophylaxis is shown in Table 2. The chemoprophylaxis group had a higher proportion of patients that were taking preoperative antiplatelets/anticoagulants (Fisher’s Exact, p = 0.001) and with a history of diabetes mellitus (Fisher’s Exact, p < 0.001).

**Table 2:** Comparison of characteristics between patients that did/did not receive chemoprophylaxis

	Chemoprophylaxis (N = 478)	No chemoprophylaxis (N = 173)	Comparison
Age (years)			
Mean	55	53	T-test, t = 1.37, p = 0.17
Range	16–86	19–86	
Gender			
Male	237/478 (50%)	89/173 (51%)	Fisher’s Exact, p = 0.72
Female	241/478 (50%)	84/173 (49%)	
Pre-op antiplatelets or anticoagulants			
No	430/478 (90%)	169/173 (98%)	**Fisher’s Exact, p = 0.001**
Yes	48/478 (10%)	4/173 (2%)	
Pre-op thrombocytopenia			
No	467/478 (98%)	166/173 (96%)	Fisher’s Exact, p = 0.28
Yes	11/478 (2%)	7/173 (4%)	
Diabetes mellitus			
No	333/478 (70%)	159/173 (92%)	**Fisher’s Exact, p < 0.001**
Yes	145/478 (30%)	14/173 (8%)	
Histology			
Pituitary adenoma	390/478 (82%)	130/173 (75%)	Chi-Squared = 13.6 p = 0.06
Cushing’s	61/478 (13%)	18/173 (10%)	
Rathke's cyst	14/478 (3%)	6/173 (4%)	
Craniopharyngioma	12/478 (3%)	1/173 (1%)	
Other tumour*	14/478 (3%)	7/173 (4%)	
Cystic lesion	8/478 (2%)	4/173 (2%)	
Inflammatory/infective	17/478 (4%)	7/173 (4%)	
Vascular (apoplexy)	7/478 (2%)	10/173 (6%)	
Non-diagnostic	16/478 (3%)	8/173 (5%)	

Bold indicates statistical significance.

### Complications

There were no cases of postoperative VTE in our series within 3 months of surgery.

Epistaxis occurred in 15/651 (2%) patients overall: 4/173 patients (2%) that did not receive chemoprophylaxis (median day 0 postoperatively, range 0–1 days), 7/478 patients (1.5%) prior to commencement of chemoprophylaxis (median day 0 postoperatively, range 0–3 days) and 4/478 patients (1%) that were already on chemoprophylaxis (median day 3 postoperatively, range 2–13 days). Thus, the overall rate of post-chemoprophylaxis epistaxis was 4/478 (1%). Epistaxis occurred a median of 1.5 days post-commencement of chemoprophylaxis (range 1–12 days) and was severe enough to with-hold chemoprophylaxis in 2 patients. Chemoprophylaxis was not associated with a statistically significant increase in epistaxis (Fisher’s Exact, p > 0.99).

6 Patients (1%) developed postoperative haematomas. 5 were treated surgically and 1 was treated conservatively. We evaluated factors predictive of haematoma formation in univariate analysis (Table 3). None of the factors evaluated were significantly associated with postoperative haematoma formation—age (p = 0.69), gender (p > 0.99), history of antiplatelet/anticoagulant usage (p > 0.99), thrombocytopenia (p > 0.99), diabetes mellitus (p > 0.99), histology (p = 0.68) and chemoprophylaxis usage (p > 0.99). Notably, five out of the six postoperative haematomas occurred in patients receiving chemoprophylaxis, which was early in 4/5 (80%).

**Table 3:** Factors predictive of postoperative haematoma formation

	Rate of haematoma formation	Univariate analysis
Age (years)		
≤ 55	4/331 (1%)	Fisher’s Exact, p = 0.69
> 55	2/320 (< 1%)	
Gender		
Male	3/326 (1%)	Fisher’s Exact, p > 0.99
Female	3/325 (1%)	
Antiplatelets or anticoagulants		
No	6/599 (1%)	Fisher’s Exact, p > 0.99
Yes	0/52 (0%)	
Thrombocytopenia		
No	6/633 (1%)	Fisher’s Exact, p > 0.99
Yes	0/18 (0%)	
Diabetes mellitus		
No	5/492 (1%)	Fisher’s Exact, p > 0.99
Yes	1/159 (1%)	
Histology		
Pituitary adenoma	5/515 (1%)	Chi-Squared = 0.18, p = 0.68
Rathke's cyst	0/20 (0%)	
Craniopharyngioma	0/13 (0%)	
Other tumour*	0/21 (0%)	
Cystic lesion	0/12 (0%)	
Inflammatory/infective	0/24 (0%)	
Vascular (apoplexy)	0/17 (0%)	
Non-diagnostic	1/24 (4%)	
Chemoprophylaxis		
No	1/173 (0.6%)	Fisher’s Exact, p > 0.99
Yes	5/478 (1.0%)	

In particular, chemoprophylaxis was not associated with a significantly increased risk of haematoma formation

## Discussion

**Fig. 1:**
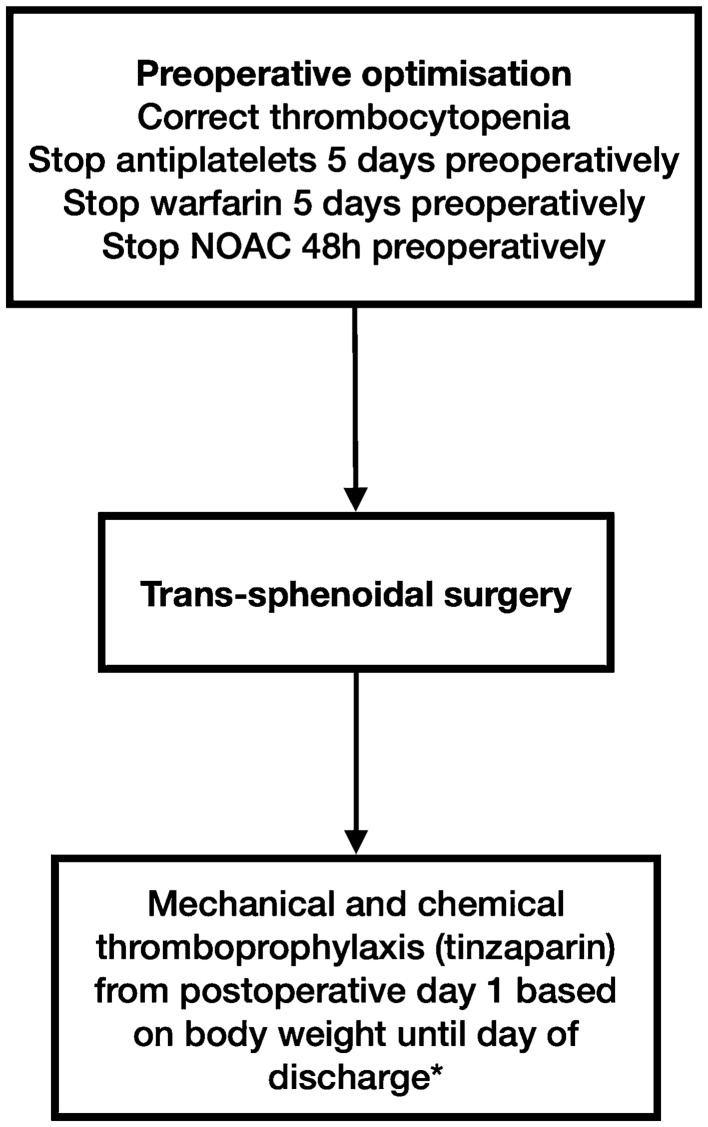
Institutional protocol for thromboprophylaxis after pituitary surgery. *Note that this decision is reviewed on a daily basis and patients who are at high risk for bleeding events may be exempt. The preferred agent for chemical thromboprophylaxis at our centre is tinzaparin, which is administered at a dose according to body weight. *NOAC* novel oral anticoagulants

In this single-centre series, we describe our experience with chemoprophylaxis in patients undergoing first time trans-sphenoidal surgery. Most patients (73%) in our study received postoperative chemoprophylaxis that was initiated on the first postoperative day in the vast majority (92%). The chemoprophylaxis group had a higher incidence of preoperative antiplatelets/anticoagulant usage and diabetes mellitus compared to patients that did not receive chemoprophylaxis. There was a low incidence of VTE (0%), epistaxis (1%) and clinically significant postoperative haematomas (1%). Although most postoperative haematomas (five out of six) occurred in patients receiving chemoprophylaxis, chemoprophylaxis was not associated with a statistically significant increased risk of postoperative haematoma formation. Our relatively aggressive strategy including combined mechanical and chemical thromboprophylaxis from postoperative day 1 appeared to be effective, for the vast majority of patients (Fig. 1).

The proportion of patients receiving chemoprophylaxis in the present study was much greater than other large neuro-oncological series describing rates of 30–40%, that have also found a tendency for a lower rate of chemoprophylaxis with primary brain tumours [17]. The absence of VTE in this study supports the findings of a systematic review and meta-analysis which showed that chemoprophylaxis significantly decreased the risk of both asymptomatic and symptomatic VTE in patients after neurosurgical intervention [7]. This finding is even more significant given that 12% of our cohort had Cushing’s disease, which has previously been identified as a risk factor for thrombosis, with several authors recommending postoperative chemoprophylaxis in this group [18]. Indeed studies have demonstrated a pro-thrombotic state in untreated Cushing’s disease that can lead to a high incidence of postoperative VTEs when institutional protocols do not routinely administer chemoprophylaxis. For example, Manetti et al. reported their experience with 40 patients—36 of which had Cushing’s disease and 4 of which had adrenal adenomas, undergoing trans-sphenoidal and adrenal surgery. They did not routinely commence chemoprophylaxis and VTE was noted in 2/40 (5%) patients postoperatively, one at 2 days and one at two months postoperatively [19]. Our approach resulted in fewer cases of perioperative thromboses compared to this and prior operative series including similar numbers of patients with Cushing’s disease [20]. Some authors recommend an even more aggressive approach, continuing chemoprophylaxis routinely for 28 days post-procedure including after discharge, due to a median time to VTE of around 2 weeks, though these series include adrenal rather than pituitary tumours [21].

Other studies evaluating chemoprophylaxis against VTE in other neurosurgical subspecialties showed a greater incidence of VTE than in our study, though a greater proportion of patients in our study received chemoprophylaxis [22, 23]. Patients that did not receive chemoprophylaxis represented a minority of included patients and the zero incidence of VTE may have resulted from our tendency to utilize this measure in the majority of patients. However, patients who did not receive chemoprophylaxis also did not have any VTE events and all patients had mechanical prophylaxis. The absence of VTE events in our cohort may also be attributed to the minimally invasive nature of trans-sphenoidal surgery and the fact that pituitary surgery rarely results in decreased mobility and our patients are mobilised on the same day as surgery, which likely further reduces the risk of VTE.

The overall incidence of pituitary haematoma in our study (1%) was within the range reported in previous large institutional series of 1–2% [24–26]. Thus, our findings correlate with studies across other neurosurgical subspecialties commenting that chemoprophylaxis does not significantly increase the risk of haematoma formation [7–11].

Due to the absence of VTE cases in our study, we were unable to compare early administration of chemoprophylaxis (on the first postoperative day) to delayed chemoprophylaxis (after the first post-operative day). Our practice is typically to commence chemoprophylaxis on the first postoperative day, in line with UK national guidance [6]. There is currently no clear guidance in the literature as to the benefit of early versus late chemoprophylaxis. In traumatic brain injury literature, there appears to be increasing evidence of benefit for early VTE administration [27, 28].

Limitations of the present study include its single-centre design and retrospective data extraction, albeit with interrogation of prospective databases. Due to the low incidence of haematomas and VTE identified, we could not identify any individual patient characteristics that were associated with either. Furthermore, we did not collect data on variables that are associated with VTE risk such as body mass index, ethnicity and smoking history [22, 29]. In the subgroup of patients with Cushing’s disease, studies have reported VTE events up to 3 years postoperatively in association with disease relapse, which is longer than the follow-up time in the present study [30].

## Conclusion

In this single centre study, we report our experience of chemoprophylaxis in patients undergoing elective trans-sphenoidal pituitary surgery. Chemoprophylaxis with LMWH was used in around three quarters of patients, usually from the first postoperative day. We found a 0% risk of VTE, which was substantially lower than other reports, 1% risk of epistaxis and 1% risk of postoperative haematoma formation that is within the previously reported range. We did not see any VTE complications in ACTH-dependent Cushing’s disease patients, despite the known elevated risk. In our cohort, early chemoprophylaxis from post-operative day 1 represents a safe intervention that may reduce the risk of VTE events without significantly increasing bleeding events. Our findings require further validation in larger multicenter prospective series or ideally a randomised controlled trial, to ensure safety and generalisability. There remain insufficient data for/against routine chemical thromboprophylaxis after endoscopic trans-sphenoidal pituitary surgery. However, this remains a widely used strategy in many centres worldwide as part of their postoperative care bundle to reduce VTE complications. Our data demonstrates that this is a safe approach.

## Data Availability

Not applicable.

## Code availability

Not applicable.
